# A near complete genome assembly of the East Friesian sheep genome

**DOI:** 10.1038/s41597-024-03581-w

**Published:** 2024-07-11

**Authors:** Xiaoxiao You, Qinyuan Fang, Chunhai Chen, Junwei Cao, Shaoyin Fu, Teng Zhang, Shenyuan Wang, Xiaolong He, Jiangfeng He, Yang Zhou, Biao Wang, Liwei Wang, Zheng Wang, Tianhao Sun, Xukui Yang, Rigele Te, Jianbo Jian, Huanmin Zhou, Yanfeng Dai, Yongbin Liu

**Affiliations:** 1 Inner Mongolia University, Hohhot, China; 2 BGI Genomics, Shenzhen, China; 3 Inner Mongolia Agricultural University, Hohhot, China; 4 Inner Mongolia Academy of Agricultural & Animal Husbandry Sciences, Hohhot, China

**Keywords:** Structural variation, Zoology

## Abstract

Advancements in sequencing have enabled the assembly of numerous sheep genomes, significantly advancing our understanding of the link between genetic variation and phenotypic traits. However, the genome of East Friesian sheep (*Ostfriesisches Milchschaf*), a key high-yield milk breed, remains to be fully assembled. Here, we constructed a near-complete and gap-free East Friesian genome assembly using PacBio HiFi, ultra-long ONT and Hi-C sequencing. The resulting genome assembly spans approximately 2.96 Gb, with a contig N50 length of 104.1 Mb and only 164 unplaced sequences. Remarkably, our assembly has captured 41 telomeres and 24 centromeres. The assembled sequence is of high quality on completeness (BUSCO score: 97.1%) and correctness (QV: 69.1). In addition, a total of 24,580 protein-coding genes were predicted, of which 97.2% (23,891) carried at least one conserved functional domain. Collectively, this assembly provides not only a near T2T gap-free genome, but also provides a valuable genetic resource for comparative genome studies of sheep and will serve as an important tool for the sheep research community.

## Background & Summary

Selective breeding for different agricultural purposes, such as meat, wool, and milk, have established many sheep breeds with unique characteristics worldwide^1^. The East Friesian sheep (*Ostfriesisches Milchschaf*) is a highly specialized breed. The breed originates from the Frisia region of both the Netherlands and Germany, and is considered to be the world’s highest producing dairy sheep^2,3^. In a single lactation, the East Friesian sheep can produce 500–700 kg of milk over a period of approximately 230 days^4^. Additionally, East Friesian sheep have a relatively high average number of lambs per ewe, 2.25 lambs/litter, but the carcass of lambs is very lean^5^. In physical appearance, East Friesian sheep have many unique features. They have a relatively large body, head, face, legs, ears all clean of wool. Their most distinctive physical feature is a “rat-tail” which is thin and devoid of wool. The East Friesian sheep, renowned for its adaptability, has been successfully crossbred with breeds known for their robust ketone body composition, such as Suffolk, Dorset, and Texel. This strategic crossbreeding not only enhances the meat quality of the East Friesian sheep but also ameliorates the traits of breeds that exhibit lower milk yields and suboptimal reproductive and lambing capabilities. Hailing from the northern regions of Germany and the Friesland area in the Netherlands, the East Friesian breed has garnered international attention and has been integrated into the livestock industries of various countries, including China, the United Kingdom, and South Africa. The exploration of the breed’s genetic makeup at the molecular level presents a compelling opportunity to deepen our comprehension of the genetic underpinnings of economically significant traits in sheep, thereby contributing to the advancement of the field.

*De novo* genome assembly is a fundamental and powerful tool employed in the realm of molecular research. Several genomes of sheep genomes have been made publicly available in databases, including East Friesian sheep^6^, Tibetan sheep^7^, Rambouillet sheep^8^, and Texel sheep^9^. Despite the achievement of chromosome-level assembly in these sheep genomes, there still exist unidentified regions containing gaps that require further investigation and determination. A number of assemblers have been developed for long reads assembly, such as Falcon^10^, Flye^11^, Canu^12^, wtdbg2^13^, NextDenovo^14^ and Hifiasm^15^. The Hifiasm method stands out for its utilization of string-overlap graphs to represent genomes, encode information for algorithmic analysis, and visually present both primary and alternative paths along a DNA sequence^16^. New developments in long-read sequencing technologies, such as Pacific Biosciences (PacBio) circular consensus (CCS) long-read sequencing and ultra-long ONT sequencing, has revolutionized our ability to acquire comprehensive chromosome sequences spanning from one telomere to another. With the availability of a complete genome sequence, researchers would have the opportunity to thoroughly investigate and gain a deeper understanding of genome function, regulation, and evolution^17,18^.

**Fig. 1:**
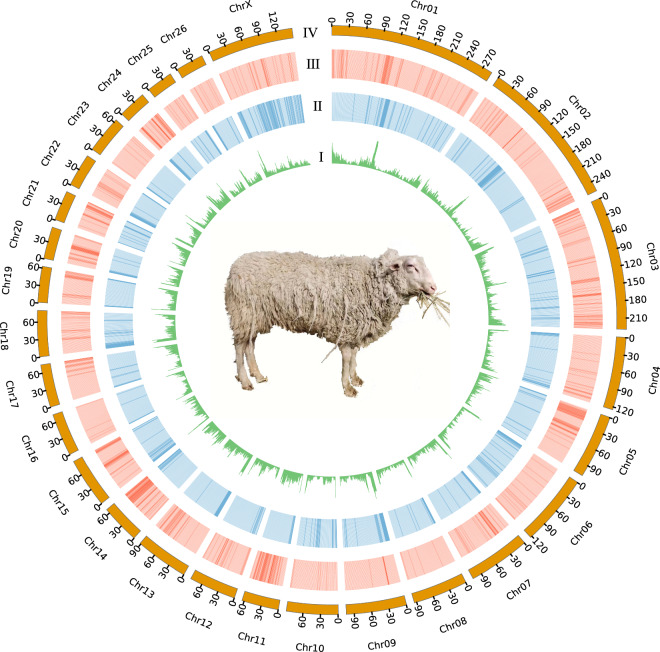
Circos plot of the EFS v2.0 genome. From inside to outside, I: GC content in nonoverlapping 1 Mb windows (histograms); II: percent coverage of repetitive sequences in nonoverlapping 1 Mb windows (heat maps); III: gene density calculated based on the number of genes in nonoverlapping 1 Mb windows (heat maps); IV: 27 super-scaffolds. Lengths are shown in Mb.

**Fig. 2:**
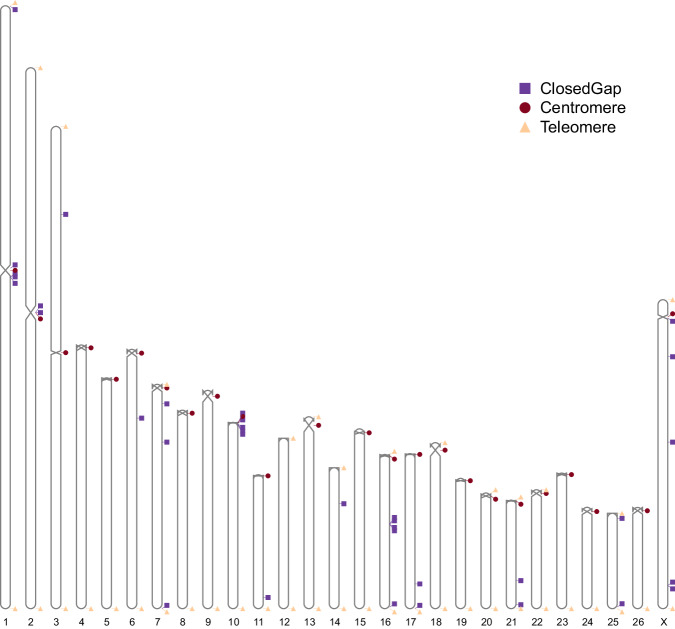
Overview of the near T2T and gap-free EFS v2.0 reference genome. The box represents the 35 closed gaps identified from GCA_018804185.1. The triangle represents the telomere region, and the circle represents the centromere region.

In this study, we present the first near T2T gap-free genome assembly for East Friesian sheep using a combination of PacBio high-fidelity (HiFi) long-read, Oxford Nanopore (ONT) ultra long-read, and high-throughput chromosome conformation capture (Hi-C) sequencing data. In total, we generated 321 Gb (~107X coverage) ONT reads with a N50 of 63.5 kb, 148 Gb PacBio HiFi CCS reads with a N50 of 22.1 kb (~49X coverage), and 396 Gb Hi-C data (MGISEQ paired-end reads, ~132X coverage) (Table 1). The final genome assembly of East Friesian sheep, termed as EFS v2.0, is about 2.96 Gb with a scaffold N50 of 104.10 Mb, comprising 27 chromosomes without any gaps (Table 2; Fig. 1) and 164 unplaced sequences. We observed that 94.53% of these unplaced sequences consist of repetitive elements, among which satellite sequences constitute 84.64%. Further research and refinement are needed to determine their precise genomic location and functional relevance. The EFS v2.0 assembly captured 41 telomeres and 24 centromeres (Table 3). Notably, the EFS v2.0 assembly closed 35 gaps in total compared to the previously published East Friesian genome^6^ (Fig. 2).

**Table 1:** Summary of sequencing data of East Friesian sheep genome.

	Flow cell number	Reads Number	Total length (GB)	Genome depth	N50 length of reads (bp)
Raw ultra-long ONT data	3	8,180,779	321	107	63,509
Clean ultra-long ONT data (>=80 kb)	3	1,017,473	108	36	103,117
Error corrected ONT data (>=80 kb)	3	1,004,276	105	35	101,717
PacBio subreads data	5	113,316,791	2,207	736	22,376
PacBio CCS data	5	6,914,516	148.3	49	22,086
Raw Hi-C data	—	2,641,764,362	396	132	150
Clean Hi-C data	—	2,631,885,258	395	132	150

**Table 2:** Comparison of four sheep genomes.

	East Friesian (EFS v2.0)	East Friesian (GCA_018804185.1)	Tibetan sheep (GCA_017524585.1)	Rambouillet (GCA_016772045.1)	Texel (GCA_000298735.2)
Genome size (Gb)	2.96	2.90	2.65	2.63	2.62
Number of scaffolds	191	937	58	142	5,466
N50 of scaffolds (bp)	104,103,076	96,203,338	105,184,753	101,274,418	100,009,711
Chromosome-scale scaffolds (bp)	2,726,314,016 (92.2%)	2,664,530,299 (91.8%)	2,649,881,505	2,615,649,360	2,584,815,894
Number of contigs	191	972	168	226	49,782
N50 of contigs (bp)	104,103,076	85,264,699	74,601,179	43,178,051	144,057
Number of Gap	0	35	110	84	44,566
Protein-coding number	24,580	not available	20,688	21,257	20,545
GC content of the genome	43.9%	43.6%	41.9%	42.0%	41.5%

**Table 3:** Centromere positions of East Friesian sheep genome.

Chromosomes ID	Chromosome length	Centromere start	Centromere end
Chr01	287,587,234	123,580,000	128,830,000
Chr02	258,028,803	113,470,000	120,150,000
Chr03	230,046,731	107,030,000	108,840,000
Chr04	125,767,300	1	2,700,000
Chr05	110,109,836	1	1,340,000
Chr06	123,671,404	1	3,560,000
Chr07	106,996,842	20,000	3,430,000
Chr08	94,674,297	170,000	2,690,000
Chr09	104,103,076	1	5,560,000
Chr10	88,865,759	1	530,000
Chr11	63,898,494	340,000	770,000
Chr12	81,322,787	—	—
Chr13	91,530,625	980,000	7,160,000
Chr14	67,246,060	—	—
Chr15	85,768,580	1,170,000	2,590,000
Chr16	73,547,930	30,000	1,270,000
Chr17	73,961,413	140,000	620,000
Chr18	79,180,959	720,000	6,400,000
Chr19	62,167,297	670,000	1,590,000
Chr20	55,174,739	440,000	2,400,000
Chr21	51,749,166	100,000	670,000
Chr22	56,726,796	280,000	3,260,000
Chr23	64,680,290	1	1,430,000
Chr24	48,344,355	630,000	3,410,000
Chr25	45,498,135	—	—
Chr26	48,284,804	1	3,170,000
ChrX	147,380,304	7,220,000	9,310,000

**Fig. 3:**
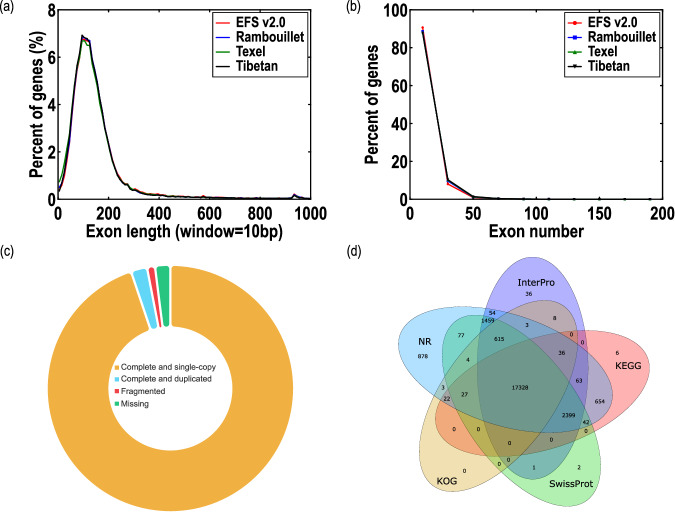
Quality assessment of the protein-coding genes in the EFS v2.0 assembly. (**a**) Comparison of exon length among four sheep gene sets. Window refers to the length of every point. (**b**) Comparison of exon number among four sheep gene sets. No obvious unexpected differences exist among these four organisms, indicating the high quality of gene structure annotation. (**c**) BUSCO assessment results of protein-coding genes in the EFS v2.0 assembly. (**d**) Gene function annotation results in a statistics Venn diagram using five public databases: NR, InterPro, KEGG, SwissProt and KOG.

**Fig. 4:**
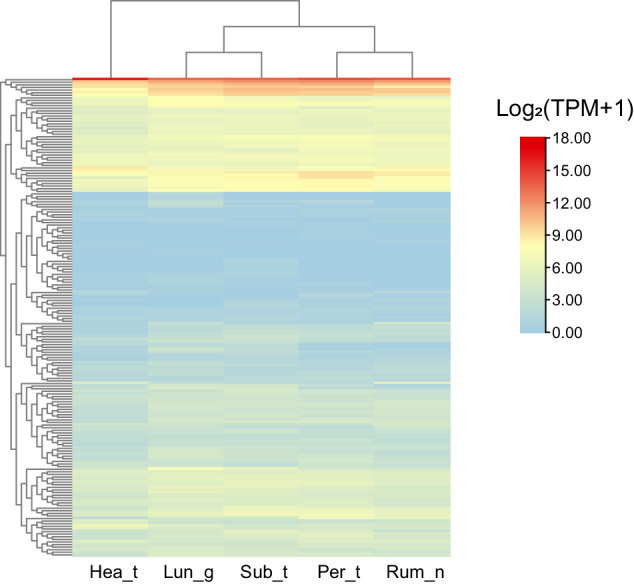
Heatmap representation of new assembled genes. Rows represent new assembled genes, and columns represent 5 different samples. The bar in the upper right corner represents log 2 transformed TPM values. Blue and red boxes represent genes showing lower and higher expression levels, respectively. “Hea_t” represents heart, “Rum_n” represents rumen, “Sub_t” represents subcutaneous fat, “Lun_g” represents lung, and “Per_t” represents perirenal fat.

In the EFS v2.0 genome, repeat sequences accounted for 1.60 Gb, representing 53.98% of the assembly (Table 4). Long interspersed nuclear elements (LINE) retrotransposons (41.46%) were the most abundant component among repetitive elements, which was consistent with a previous study^19^ (Table 5). Gene annotation identified 24,580 protein-coding genes. Of which, 24,536 genes (99.8%) were anchored to 27 chromosomes (Fig. 1), while 44 genes anchored to unplaced scaffolds. The length and number of exons were similar to those of three other sheep breeds (Fig. 3a,b). Furthermore, the predicted proteins achieved a complete BUSCO score of approximately 98%, indicating high quality annotation (Fig. 3c). 23,891 (97.2%) protein-coding genes were successfully annotated in diverse databases, including Gene Ontology (GO), KOG, Interpro, SwissProt^20^, Kyoto Encyclopedia of Genes and Genomes (KEGG)^21^, NCBI nonredundant database (NR), and Translation of European Molecular Biology Laboratory (Trembl) (Table 6). Moreover, 17,328 (~70.5%) genes were supported by all five databases (Fig. 3d). Based on transcriptomic deep-sequencing data, we investigated gene expression level in five different tissues (Table 7). A total of 15,263 (62.2%) genes showed detectable expression levels (transcripts per million ≥ 1) in one or more of these tissues. Through structural variants analysis with the previously published East Friesian sheep^6^, we identified 232 newly assembled genes, among which 151 were expressed in 5 different transcriptome samples (Table 8; Fig. 4).

**Table 4:** General statistics of repeats in the EFS v2.0 assembly.

Type		Repeat Size	% of genome
**Tandem repeats**		121,015,125	4.09
**Interspersed repeats**			
Repeatmasker		965,987,732	32.67
Proteinmask		555,689,821	18.80
*De novo*		1,460,536,811	49.40
**Total**		1,596,071,511	53.98

Note: Some elements may partially overlap with another element domain.

**Table 5:** Transposable elements (TEs) in the assembled EFS v2.0 assembly.

Type	Repbase TEs		TE protiens		*De novo*		Combined TEs	
	Length (Bp)	% in genome	Length (Bp)	% in genome	Length (Bp)	% in genome	Length (Bp)	% in genome
DNA	26,247,775	0.89	2,137,907	0.07	271,885	0.01	26,477,799	0.90
LINE	748,843,603	25.33	546,714,112	18.49	1,125,752,351	38.08	1,225,790,468	41.46
SINE	133,041,949	4.50	0	0.00	563,878	0.02	133,585,032	4.52
LTR	76,620,857	2.59	6,905,477	0.23	127,898,467	4.33	196,780,336	6.66
Other	233	0.00	0	0.00	0	0.00	233	0.00
Unknown	0	0.00	0	0.00	700,079	0.02	700,079	0.02
Total	965,987,732	32.67	555,689,821	18.80	1,199,035,229	40.56	1,300,301,311	43.98

Note: This statistical table does not contain Tandem Repeats, some elements may partly include another element domain.

*Combined: the non-redundant consensus of all repeat prediction/classification methods employed.

^†^Unknown: the predicted repeats that cannot be classified by RepeatMasker;

LINE, long interspersed nuclear elements; SINE, short interspersed nuclear elements; LTR, long terminal repeat.

**Table 6:** Number of functional annotations for predicted genes in the EFS v2.0 assembly.

Type		Gene number	Percentage
**Total**		24,580	100%
**Nr**		23,664	96.27%
**Swissprot**		21,954	89.32%
**KEGG**		20,577	83.71%
**KOG**		18,046	73.42%
**TrEMBL**		23,755	96.64%
**Interpro**	All	22,002	89.51%
	GO	16,578	67.45%
**Annotated**		23,891	97.20%
**Unannotated**		689	2.80%

**Table 7:** Summary of RNA-seq sequencing data of East Friesian sheep genome.

Sample	Raw reads	Raw bases	Clean reads	Clean bases	Q20 (%)	Q30 (%)
Lun_g	65,206,128	9,780,919,200	61,666,246	9,249,936,900	97.86	92.96
Rum_n	74,652,518	11,197,877,700	70,628,290	10,594,243,500	98.02	93.47
Sub_t	77,938,068	11,690,710,200	63,021,986	9,453,297,900	98.04	93.57
Per_t	69,699,584	10,454,937,600	66,091,478	9,913,721,700	97.95	93.25
Hea_t	66,270,030	9,940,504,500	62,984,582	9,447,687,300	97.94	93.19

Note: “Hea_t” represents heart, “Rum_n” represents rumen, “Sub_t” represents subcutaneous fat, “Lun_g” represents lung, and “Per_t” represents perirenal fat.

**Table 8:** The improvement of EFS v2.0 assembly.

Chromosomes	EFS v2.0 Length (bp)	GCA_018804185.1 Length (bp)	EFS v2.0 gap numbers	GCA_018804185.1 gap numbers	EFS v2.0 Gene number	EFS v2.0 New assembled genes
1	287,587,234	284,533,122	0	5	2,411	19
2	258,028,803	254,299,457	0	2	1,792	15
3	230,046,731	229,824,859	0	1	2,400	24
4	125,767,300	121,425,072	0	0	847	9
5	110,109,836	108,581,918	0	0	1,380	5
6	123,671,404	118,750,793	0	1	705	16
7	106,996,842	104,409,019	0	3	1,034	11
8	94,674,297	91,893,355	0	0	552	8
9	104,103,076	96,203,338	0	0	564	8
10	88,865,759	88,811,564	0	5	438	6
11	63,898,494	63,197,725	0	1	1,304	7
12	81,322,787	81,831,065	0	0	727	7
13	91,530,625	85,264,699	0	0	852	5
14	67,246,060	67,917,403	0	1	1,379	13
15	85,768,580	85,099,227	0	0	1,036	12
16	73,547,930	73,688,838	0	5	383	5
17	73,961,413	74,056,358	0	2	624	5
18	79,180,959	73,405,259	0	0	624	6
19	62,167,297	60,536,646	0	0	589	4
20	55,174,739	52,767,035	0	0	800	6
21	51,749,166	52,149,183	0	2	714	4
22	56,726,796	51,710,764	0	0	434	4
23	64,680,290	62,896,507	0	0	359	4
24	48,344,355	45,761,874	0	0	798	4
25	45,498,135	45,186,384	0	2	334	3
26	48,284,804	45,990,704	0	0	264	6
X	147,380,304	144,338,131	0	5	1,192	16
Total	2,726,314,016	2,664,530,299	0	35	24,536	232

## Methods

### Sample collection, DNA preparation and RNA extractions

A 1-year-old female East Friesian sheep from Inner Mongolia key Lab of Bio-manufacture in Inner Mongolia autonomous region of China was chosen for DNA and RNA sequencing. The assembled sequence does not include the Y chromosome due to sampling from females. The animal was healthy, and no genetic defects were observed in it or its parents.

DNA was extracted from fresh blood specimen using the QIAGEN Blood & Cell Culture DNA Midi Kit according to the manufacturer’s instruction (QIAGEN, Germany). TRIzol (Invitrogen, Carlsbad, CA, United States) was used to extract total RNA from heart, rumen, subcutaneous fat, lung and perirenal fat tissues. The concentration of total RNA was determined using the Nano 6000 spectrophotometer Assay Kit of the Bioanalyzer 2100 system (Agilent Technologies, Santa Clara, CA, United States). The RNA purity was determined using the Qubit® RNA Assay Kit in a Qubit® 2.0 Fluorometer (Life Technologies, Camarillo, CA, United States).

### Long insert libraries preparation and sequencing

The library construction and sequencing of RNA-seq full-length transcripts were conducted using a method similar to that described in Yuan, Ge *et al*.^22^, resulting in 437,807 full-length non-chimeric reads with mean length of 1,388 bp.

For the DNA PacBio long inserts libraries, the preparation was carried out in accordance with the “Using SMRTbell Express Template Prep Kit 2.0 With Low DNA Input” protocol^23^ provided by PacBio (Pacific Biosciences, USA). This resulted in libraries with an insert size of approximately 20 kb. Subsequently, the libraries were subjected to sequencing using PacBio Sequel II platforms operating in CCS mode. The subreads were processed through the CCS algorithm of SMRTLink (v8.0.0)^24^ with specific parameters: “-minPasses 3 -minPredictedAccuracy 0.99 -minLength 500”, yielding 148 Gb of PacBio’s long high-fidelity (HiFi) reads in total.

Furthermore, ultra-long DNA ONT libraries were created following the protocols detailed by Shafin *et al*.^25^. These libraries were then sequenced on the PromethION sequencer platform (Oxford Nanopore Technologies, UK). The sequencing effort resulted in the production of 8,180,779 reads, with an N50 value of 63,509 bp.

### Short insert libraries preparation and sequencing

RNA-seq libraries were prepared uisng the NEBNext® Ultra™ RNA Library Prep Kit for Illumina® (NEB, Ipswich, MA, USA) following the manufacturer’s protocol. Subsequently, the RNA libraries were sequenced on a MGISEQ-2000 platform, producing 150 bp paired-end reads.

The Hi-C library was prepared using the same method described in Yin, Chen *et al*.^26^ with the same blood specimen and sequenced on a MGISEQ-2000 instrument. A total of 395 Gb of clean data were obtained from 396 Gb of sequencing data using software SOAPnuke (v2.0)^27^ with parameters “-n 0.01 -l 20 -q 0.1 -i -Q 2 -G 2 -M 2 -A 0.5”.

### Genome assembly

With the HiFi reads, the primary contigs were assembled using Hifiasm (v 0.16.1)^15^ with default parameters. The Hi-C valid reads were employed to anchor contigs onto chromosomes through Juicer^28^ and 3d-dna pipeline^29^. The chromosome nomenclature was adopted for the chromosome numbering on the basis of their collinearity with 27 chromosomes of Texel sheep genome^30^. To achieve a near T2T gap-free reference genome assembly, gaps in the assembly genome were filled using LR_Gapcloser^31^ with error-corrected ONT long reads produced by NECAT^32^.

### Annotation of repetitive sequences and identification of telomeres and centromeres

Two strategies, *de novo* and homolog methods, were applied to annotate repetitive sequences. RepeatModeler (v1.0.4)^33^ was used to identify *de novo* repeats, and LTR-FINDER (v1.0.7)^34^ was utilized to annotate long terminal repeats. DNA and protein transposable elements (TEs) were detected by RepeatMasker (v4.0.7)^35^ and RepeatProteinMasker (v4.0.7), respectively, based on Repbase database^36^. At last, tandem repeats were identified using Tandem Repeat Finder (v4.10.0)^37^. Referencing the methods described in sweet tea^38^, wild blueberry^39^, and rapeseed studies^40^, the telomeric sequences in the EFS v2.0 genome assembly were identified using quarTeT (v1.0.3)^41^ with the “-c animal” option. The quarTeT program comprises four modules: AssemblyMapper, GapFiller, TeloExplorer, and CentroMiner, of which, TeloExplorer is specifically utilized for identifying candidate telomeres. The telomere repeat monomer identified by quarTeT was “TTAGGG/CCCTAA”. The Centromics software (https://github.com/ShuaiNIEgithub/Centromics) was employed for centromere identification. Furthermore, we aligned the human rRNA sequences against the EFS v2.0 genome by using BLASTN with an E value of 1 × 10^−5^ to investigate the ribosomal DNA (rDNA) in EFS v2.0 genome. In total, we identified 1329 ribosomal RNAs (rRNAs), including 122 18S rRNA, 586 28S RNA, 59 5.8S rRNA, and 562 5S RNA.

### Protein-coding genes prediction and functional annotation

Protein-coding genes in the EFS v2.0 assembly were predicted using a similar method to Fang, Mou *et al*.^42^. For the RNA-seq-based prediction approach, clean RNA-seq reads were aligned to the EFS v2.0 assembly via Hisat2 (v2.1.0)^43^ with parameters including–sensitive–no-discordant–no-mixed -I 1 -X 1000–max-intronlen 1000000. Additionally, 437,807 full-length transcripts were matched against the EFS v2.0 assembly using GMAP (v 2017-11-15)^44^. The aligned reads were then assembled using Stringtie (v1.3.5)^45^ using the parameters: -f 0.3 -j 3 -c 5 -g 100 -s 10000. Subsequently, TransDecoder (v5.5.0) (https://github.com/TransDecoder/TransDecoder) was invoked to identify the coding sequence with default settings. In the homolog prediction method, GeMoMa (v1.9)^46^ was used to detect homologous peptides across four mammalian genomes, including human (GCA_000001405.29)^47^, Texel sheep (GCA_000298735.2)^9^, Tibetan sheep (GCA_017524585.1)^7^, and Rambouillet sheep (GCA_016772045.1)^8^. Genes that had RNA-seq-based prediction support with correct structure, but were not identified via homology-based prediction, were incorporated into the gene set. Ultimately, untranslated regions and alternative splicing regions were determined using Program to Assemble Spliced Alignment^48^. The integrated gene set was translated into amino-acid sequences and functionally annotated by mapping against KEGG^49^, Swiss-Prot^20^, TrEMBL^20^, KOG^50^, InterPro^51^ and NR (NCBI Non-redundant protein) databases using BLAST (v2.2.26)^52^ with an E-value threshold of 1E-5. Protein domains and motifs were annotated using InterProScan^53^, from which GO Ontology (GO)^54^ was derived.

### Gene expression analysis

Quality control of raw RNA-seq reads was conducted using SOAPnuke (v2.0)^15^. Afterwards, the clean reads were aligned to the EFS v2.0 genome using Hisat2 (v2.1.0)^43^, with the following parameters: ‘--phred33 -p 5 --sensitive --no-discordant --no-mixed -I 1 -X 1000’. A read count matrix was generated using featureCounts^55^. Gene expression levels were calculated using the transcripts per million (TPM) method.

### Identification of new assembled genes

The software Syri (v1.6.3)^56^ was employed to detect structural variations between the EFS v2.0 genome assembly and the previously published East Friesian sheep^6^. A gene was classified as newly assembled if the previously published East Friesian sheep^6^ exhibited a deletion of at least 50 bp and the gene region had a minimum overlap of 30% with that region.

### Reads coverage analysis of genome assembly

We assessed whether the long sequencing reads extended across the regions that required gap filling. Prior to this process, the genome contained eight gaps. We employed minimap2^57^ (v 2.24) to map both the ONT and HiFi reads to the EFS v2.0 genome. Utilizing SAMtools^58^ (v 1.10) with the ‘-q 20’ option, we filtered out low-quality and multi-mapping reads. Subsequently, we utilized the IGV software for visualizing the high-quality alignment results.

### Quality value (QV) calculations

In the realm of whole-genome sequencing, the Quality Value (QV) emerges as an essential metric for gauging the precision of nucleotide identification. The QV is derived from the Phred quality score, a measure that captures the negative logarithm of the likelihood that a given base call is erroneous. The QV is precisely calculated through the equation *QV* = −10 × log_10_ (error probability). For instance, an error probability of 0.001 equates to a QV of 30, indicating a high confidence in the correctness of the base call. Throughout the sequencing process, each nucleotide is appraised with a Phred score that is contingent upon the signal-to-noise ratio; this score is subsequently converted to a QV, thereby providing an index of the sequencing data’s fidelity. In this study, we have employed the Merqury^59^ software to meticulously compute the QV, ensuring robust data quality assessment.

## Data Records

The DNA sequence reads of East Friesian sheep (Experiment of DNA sequencing data from ultra-long ONT library: SRR26273756^60^; Experiments of DNA sequencing data from Hi-C library: SRR26273763^60^; Experiments of DNA sequencing data from PacBio HiFi library: SRR26273762^60^) and RNA sequence reads of East Friesian sheep (Experiment of 5 transcriptome libraries: SRR26273757-SRR26273761^60^) have been deposited in the Sequence Read Archive (SRA). The genome assembly have been deposited in the GenBank database under the accession number JAWMPZ000000000^61^. The files of the gene structure annotation, repeat predictions and gene functional annotation have been deposited at Figshare database^62^.

## Technical Validation

Multiple methods were employed to validate the accuracy and completeness of EFS v2.0 assembly.

**Fig. 5:**
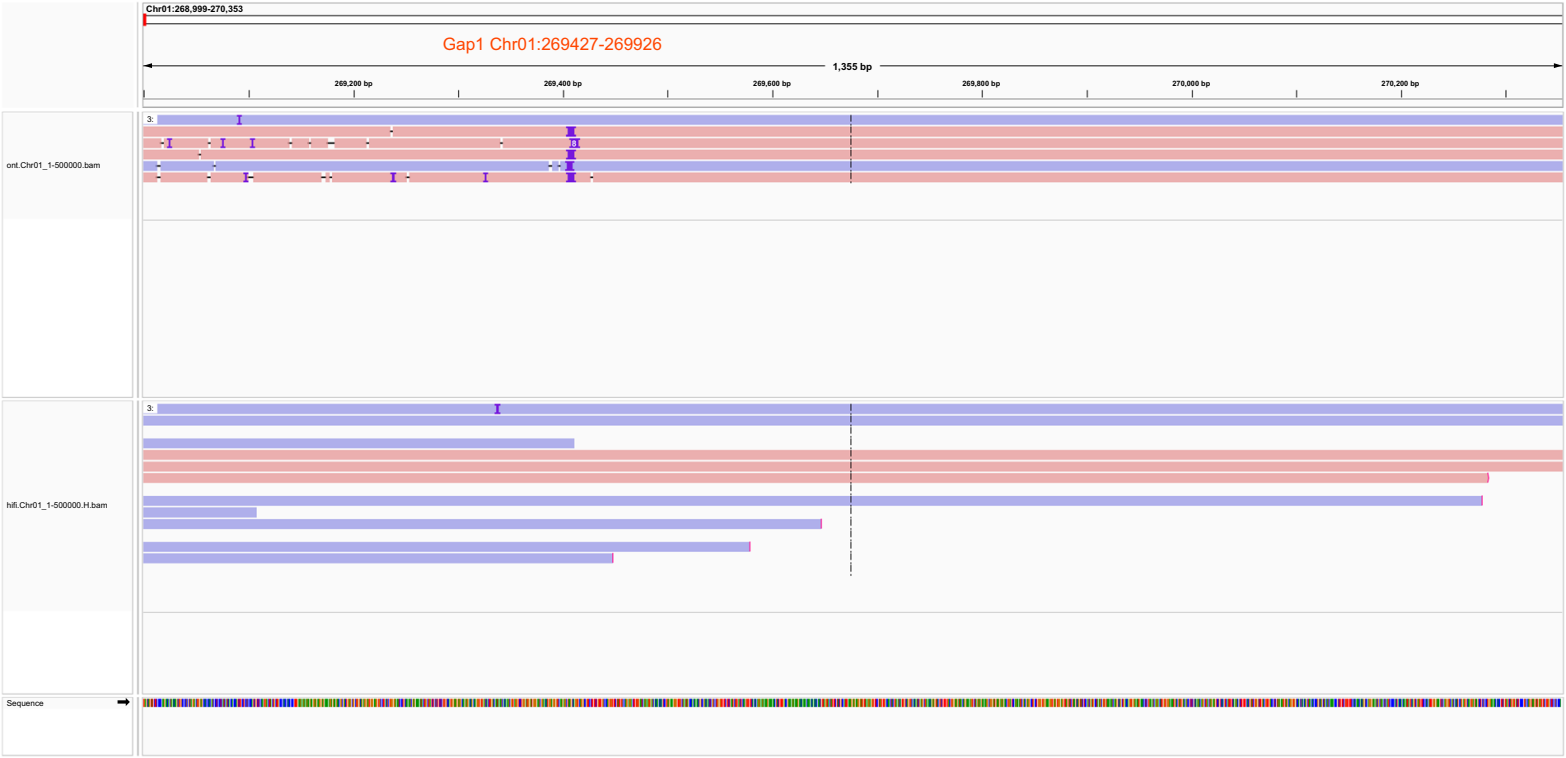
Using IGV to demonstrate the coverage of ONT and PacBio reads in the gap 1 region. The IGV images for Gap 1 through Gap 8 are available through the Figshare database^62^.

**Fig. 6:**
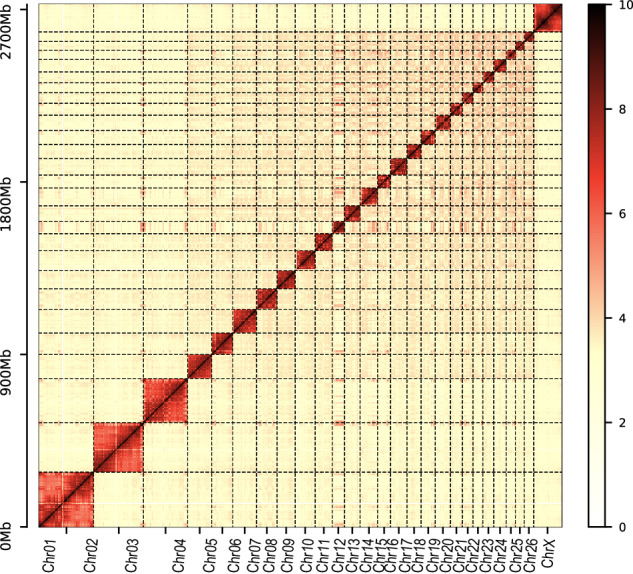
The accuracy and completeness of the EFS v2.0 genome assembly. Whole-genome Hi-C heatmap of EFS v2.0 within and between 27 chromosomes.

**Fig. 7:**
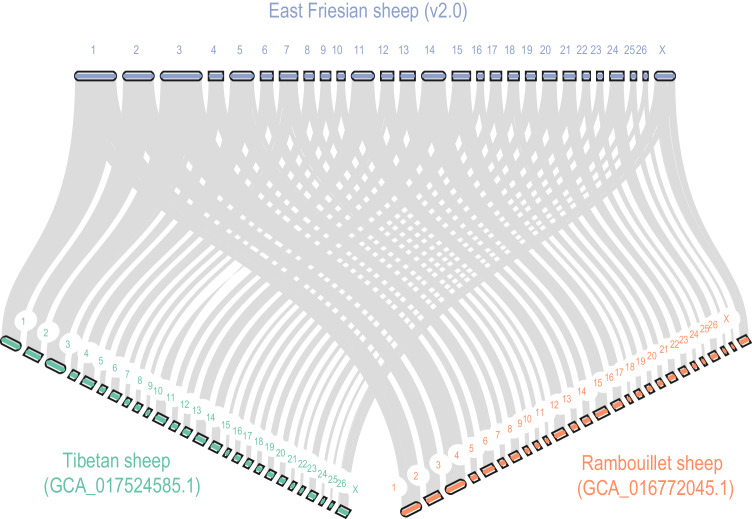
The identification of syntenic regions for EFS v2.0, Rambouillet sheep and Tibetan sheep was based on conducting homology searches using MCScan (Python version)^63^, with a minimum requirement of 30 genes per block. Macrosynteny connecting blocks of >30 one-to-one gene pairs are shown.

**Fig. 8:**
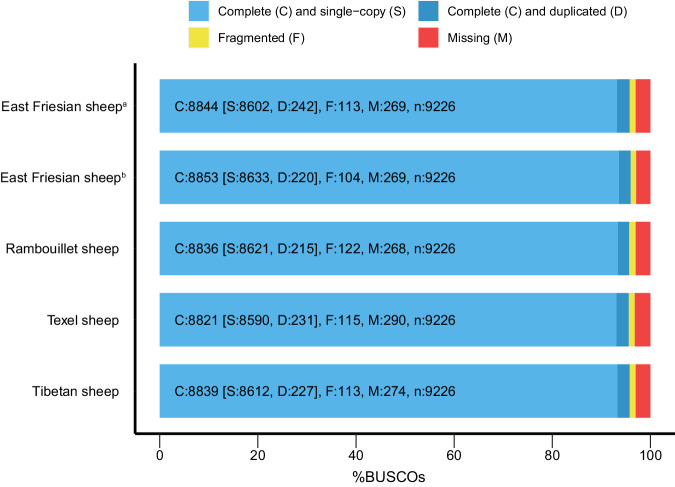
BUSCO plot of the several sheep genomes. C: Complete BUSCOs; S: Complete and single-copy BUSCOs; D: Complete and duplicated BUSCOs; F: Fragmented BUSCOs; M: Missing BUSCOs; n: Total BUSCO groups searched. East Friesian sheep^a^: GCA_018804185.1; East Friesian sheep^b^: EFS v2.0.

Firstly, we utilized long sequencing reads to ascertain their extension across the eight gap regions (Table 9). The resulting plots confirmed comprehensive coverage of the targeted regions (Fig. 5). Secondly, the Hi-C heatmap displayed high consistency across all chromosomes, demonstrating the correct ordering and orientation of contigs in the EFS v2.0 assembly (Fig. 6). Thirdly, the EFS v2.0 assembly exhibited high collinearity with Rambouillet sheep (GCA_016772045.1)^8^, Tibetan sheep (GCA_017524585.1)^7^ and the previously published East Friesian sheep (GCA_018804185.1)^6^ (Fig. 7). Fourthly, the accuracy was confirmed by the high mapping rates of two type sequences on the EFS v2.0 assembly, with 99.93% of ONT reads and 100% of HiFi reads aligning to the EFS v2.0 assembly. Notably, the sequencing assembly attained a remarkable quality value (QV) score of 69.1, signifying an exceptionally low error rate of approximately 1.26 errors per 100 million bases. This level of sequencing accuracy and reliability is highly commendable and will undoubtedly facilitate subsequent genetic analysis and research. Lastly, the Benchmarking Universal Single-Copy Orthologs (BUSCO) test revealed that EFS v2.0 assembly successfully identified 97.1% of 9,226 mammalia gene sets, which exhibiting the highest level of BUSCO completeness among the four commonly used genomes (Fig. 8).

**Table 9:** The location of the gap to be filled.

Chromosome ID	Start	End
Chr01	269,427	269,926
Chr01	391,274	391,773
Chr11	43,820,237	43,846,318
Chr11	63,558,411	63,558,809
Chr11	63,647,361	63,647,859
Chr20	33,593,787	33,599,607
Chr24	41,820,199	41,820,522
Chr25	2,393,086	2,394,781

## Data Availability

No specific code was developed for this study. The data analyses were conducted following the manuals and protocols provided by the developers of the relevant bioinformatics tools, which are described in the Methods section along with the versions used.
