# Author Correction: A near complete genome assembly of the East Friesian sheep genome

**DOI:** 10.1038/s41597-024-03755-6

**Published:** 2024-08-29

**Authors:** Xiaoxiao You, Qinyuan Fang, Chunhai Chen, Junwei Cao, Shaoyin Fu, Teng Zhang, Shenyuan Wang, Xiaolong He, Jiangfeng He, Yang Zhou, Biao Wang, Liwei Wang, Zheng Wang, Tianhao Sun, Xukui Yang, Rigele Te, Jianbo Jian, Huanmin Zhou, Yanfeng Dai, Yongbin Liu

**Affiliations:** 1 Inner Mongolia University, Hohhot, China; 2 BGI Genomics, Shenzhen, China; 3 Inner Mongolia Agricultural University, Hohhot, China; 4 Inner Mongolia Academy of Agricultural & Animal Husbandry Sciences, Hohhot, China

**Keywords:** Structural variation, Zoology

Correction to: *Scientific Data* 10.1038/s41597-024-03581-w, published online 11 July 2024

In this article the affiliation details for Chunhai Chen, Xukui Yang, and Jianbo Jian were incorrectly given as ‘Inner Mongolia Academy of Agricultural & Animal Husbandry Sciences, Hohhot, China’ but should have been ‘BGI Genomics, Shenzhen, China’. In addition, the affiliation details for Shaoyin Fu, Xiaolong He, Jiangfeng He, Biao Wang, Liwei Wang, Rigele Te, and Yongbin Liu were incorrectly given as ‘BGI Genomics, Shenzhen, China’ but should have been ‘Inner Mongolia Academy of Agricultural & Animal Husbandry Sciences, Hohhot, China’. The original article has been corrected.

